# Perception of Paediatricians and Families about Nutritional Supplements: Acceptance, Tolerability and Satisfaction in Malnourished Children (PerceptiONS Jr Study)

**DOI:** 10.3390/nu16152475

**Published:** 2024-07-30

**Authors:** María del Mar Tolín Hernani, María del Carmen Miranda Cid, María Guerrero Cuevas, Guillermo Álvarez Calatayud, The PerceptiONS Jr Study Group, César Sánchez

**Affiliations:** 1 Digestive, Hepatology and Paediatric Nutrition Unit, Infant Maternal Hospital, Hospital General Universitario Gregorio Marañón, 28007 Madrid, Spain; 8marth81@gmail.com (M.d.M.T.H.); mcmirandina@gmail.com (M.d.C.M.C.); galvarezcalatayud@gmail.com (G.Á.C.); 2 Abbott Laboratories, 28050 Madrid, Spain; maria.guerrero1@abbott.com

**Keywords:** acceptance, adherence, tolerability, satisfaction, quality of life, malnutrition, nutritional supplements, children

## Abstract

Background: Malnutrition is a common situation in the Spanish paediatric population. Malnourished children may benefit from different strategies, including dietary modifications or nutritional supplements (NS). It is important to know the different factors that can influence treatment tolerance and adherence, and their impact on nutrition monitoring. Objectives: To explore the perception of doctors who prescribe nutritional supplements (NS) in children and to investigate different factors involved in tolerance and adherence. Material and methods: A cross-sectional, descriptive observational study based on an ad hoc electronic survey designed to study doctors’ perceptions of at least five of their children with NS and their families, subjected to outpatient follow up. Variables included were the socio-demographic variables of the doctors and children, nutritional status of the patients, amount and characteristics of NS (hyper-caloric oral with fibre (HOFF), oral peptide (OPF) and hyper-caloric infant (HIF)), route of administration, perceived benefits, satisfaction, palatability, adherence, and acceptance. Results: 815 patients aged 0–16 years (mean 10.6 years; SD 7.8) were included. A proportion of 64% received HOFF, 16% FOP, and 20% HIF. A proportion of 84% received exclusive oral NS. Total daily calorie intake prescribed with NS ranged from 30–75% in 48.2% of cases, though it was significantly higher in children under 6 months of age. Improvement in overall condition, nutritional status and quality of life was observed in 82%, 79.5%, and 80% of subjects. Improvement in tolerance and digestive symptoms was reported in 83.5% and 72% of subjects. The degree of satisfaction and acceptance of NS was very good in 80% of cases, with taste being the most influential factor (82.3%). Adherence was adequate in more than 60%, and smell was the most significant feature in lack of adherence (55%). The flavour of the best-accepted supplement was chocolate (44%). A total of 97% of prescribing doctors would recommend the same formula again. Conclusions: In our study, prescribing doctors and families perceived an excellent benefit from the use of the prescribed formulas, improved quality of life, high satisfaction, acceptance, and adherence. The positive factors for adequate adherence were sufficient information about the formulations and their benefits, and continuity of care during follow-up. Prescribing doctors would recommend supplement use again given the perceived benefits and satisfaction.

## 1. Introduction

Disease-related malnutrition in children is a relatively common condition in our environment, with prevalences that vary according to different studies and that are difficult to understand [1]. Figures of up to 30% have been recorded in the hospitalised paediatric population according to the DHOSPE study [2,3].

This nutritional status determines the course, complications, severity, and hospital stay of inpatients, as well as mortality in various clinical situations and scenarios, as it influences the immune and inflammatory status and the patient’s capacity for recovery. All of these conditioning factors have led to research into new methods for early detection in order to start treatment in early onset [4,5].

In addition to the above, disease-related malnutrition entails an increase in financial health costs in the hospital stay, which is longer in these patients; an increase in drug costs, specifically antibiotic therapy; and increases in treatments at discharge, referrals to other centres, home care, readmissions and in mortality [6].

Therefore, the early detection and treatment of malnutrition in children is essential, and may benefit from different strategies, including dietary changes or nutritional supplements (NS). It is very important to understand the factors that may influence tolerance and adherence to nutritional supplements such as amount, type, duration, palatability, and accessibility, and their impact on nutrition monitoring, as these will influence patient progress in the long-term, and may change the course of their underlying disease and complications.

The objective of this study was to explore the perception of paediatricians that prescribe nutritional supplements in children, supported by information provided by families and patients and with regard to the different factors involved in tolerance, adherence, and symptom improvement with the prescribed nutritional therapy, differentiating according to type of supplement and nutritional status.

## 2. Materials and Methods

PerceptiONS Jr is a cross-sectional, descriptive, observational multi-centre study based on an ad hoc electronic survey designed to explore doctors’ perceptions of at least five of their children with malnutrition during their outpatient follow-up. 

The study was conducted by a committee of three paediatricians who are experts in the nutritional management of sick children. All committee members work in a tertiary hospital belonging to the Spanish public health system.

### 2.1. Study Participants

The electronic survey was completed by paediatricians working in the Spanish public health system who were selected for their experience in the nutritional treatment of children and who belong to various paediatric subspecialties. They gave their consent to participate in the study. The doctors were invited to participate by the study sponsor through its commercial network (Abbott Paediatric Medical Nutrition, Spain).

When agreeing to be included in the study, the participants were provided with a personal login code and access link to the electronic questionnaire, which was developed on a website specifically designed for the project and included the informed consent. The questionnaire was available for entering personal and patient data from January to November 2023. 

### 2.2. Electronic Survey

Specific content was developed for the survey by the expert committee based on previous published studies on malnutrition in children of various ages, as well as similar studies conducted in adults on the use of nutritional supplements [7,8].

This survey assessed the perception of certain parameters of the prescribing doctor in five patients with malnutrition treated with nutritional supplements.

The inclusion criteria were paediatricians who prescribed nutritional supplements to any malnourished children at risk of malnutrition due to underlying chronic disease. The exclusion criteria encompassed palliative patients (<6 months of life expectancy), patients who had received nutritional support in the past 3 months, patients with total or partial parenteral nutrition, and patients with nutritional treatment for specific diseases (inborn errors of metabolism, ketogenic diets, paediatric IBD nutritional treatment, etc.).

The same survey collected sociodemographic data of the prescribing doctors (age, sex, years of experience, and paediatric subspecialty), as well as the patients (sex, age), nutritional status, the amount and characteristics of the oral nutritional supplement (ONS) received [hypercaloric oral formula with fibre (HOFF), oral peptide (OPF), and hypercaloric infant (HIF)], and route of administration.

The nutritional status of the children was defined by applying the Waterlow nutritional index for weight ((current weight/P50 for height) × 100) to classify acute malnutrition or wasting (>100% overweight—obesity, 90–100%—normal, 80–90%—mild malnutrition, 70–80%—moderate malnutrition, and <70%—severe malnutrition) and the Waterlow index for height (current height/P50 height) × 100) for the classification of chronic malnutrition or stunting (>95%—normal, 90–95%—mild malnutrition, 85–90%—moderate malnutrition, and <85%—severe malnutrition) [2]. The anthropometric measurements were established according to the data and percentile zscore available for the Spanish population.

A second part of the survey assessed the perceived benefits of ONS, patient satisfaction and ONS acceptability, patient adherence to the prescribed supplement and its associated factors, and improvement in physical condition with the prescribed ONS. Lastly, patients were asked about their perceptions of the taste of the formula. Adherence and acceptance were assessed by the parents, and were defined as the percentage of prescribed formula that the child actually consumed (very low: <25%; low: 25 to 50%, good: 51 to 75%, very good: >75% of the prescribed treatment).

Different statistical correlations were made with baseline nutritional status, adherence, and tolerability of the patients as perceived by their parents or guardians.

The survey included closed-ended multiple choice-type questions and other Likert scale assessment questions (escale from 1 to 5), as well as some open-ended items.

### 2.3. Statistical Analysis

The software used for the statistical analysis was the SAS system version 9.4 (SAS Institute Inc., Cary, NC, USA). Categorical variables were described as total number and percentage. Continuous variables were expressed using measures of central tendency and dispersion. Categorical variables were compared using Fisher’s exact test and continuous variables were compared using Student’s t test in cases of two groups or using ANOVA test in cases of three or more groups. If the application conditions were not met, the Mann–Whitney U test or the Kruskal–Wallis test, respectively, were used.

## 3. Results

The surveys provided by 183 prescribing paediatricians were analysed (Table 1); 163 completed the study, resulting in the collection of data from 815 of the included patients and families (89% completion).

A total of 55% of patients were male. The distribution by age group was 7.6% between birth and 6 months, 9% between 6 months and 1 year, 24% between 1 and 3 years, 18.6% between 3 and 6 years, 17.3% between 6 and 10 years, and 23.5% over 10 years. A proportion of 40% of the children had mild acute malnutrition, 25% moderate acute malnutrition, and 7.4% severe malnutrition according to the Waterlow index for weight. In the case of chronic malnutrition according to the Waterlow index for height, 35% were mild, 17% moderate, and 7% severe (Table 2). A proportion of 64% of the children received HOFF, 16% OPF, and 20% HIF (Table 3).

A total of 84% of patients received supplementation by oral route only, 6% by gastrostomy, 5% by exclusive nasogastric tube (NG) and 5% via a mixed route. Use of an NG tube in children between 0 and 6 months of age was significantly higher (*p* < 0.05).

With regard to the total daily calorie intake (TCI) provided by the supplement administered with respect to the recommended daily intake (RDA) in children, 48.2% of patients received 30–79% of their TCI, 37.5% less than 30% of their TCI, and 14% received more than 80% of their TCI, with the highest total calorie percentage provided in children under one year of age (*p* < 0.05).

As regards benefits perceived by families, improvement in overall condition was observed in 82% of children, improvement in mood in 69%, vitality in 76%, improvement in symptoms in 77%, improvement in nutritional status in 79.6%, and improvement in quality of life in 79.5%, the latter two being more significant in children under 6 months of age (*p* < 0.05). Both the perceived improvement in overall condition of the child and their quality of life and achievement of the nutritional goal were significantly correlated to a greater amount of formula administered (*p* < 0.05). The benefits observed by families with the use of HOFF showed a statistically significant association between improvement in nutritional status and mood and overall condition with a higher calorie intake (*p* < 0.05). In the case of OPF, improvement in quality of life and improvement in symptoms were also observed in older children (*p* < 0.05). These changes were not confirmed in the case of HIF. None of the formulations used showed that the benefits obtained were correlated with patient age or baseline nutritional status (Table 4).

**Figure 1:**
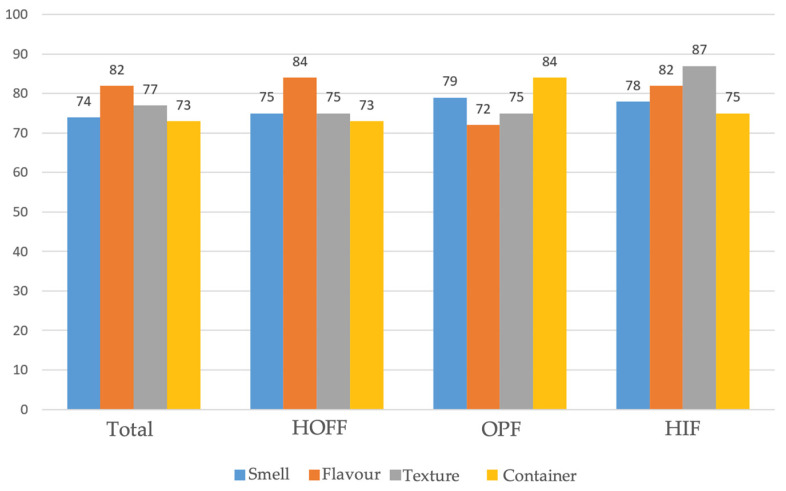
Impact of organoleptic characteristics on enteral nutrition adherence (percentage of favourable responses).

As regards satisfaction and acceptability, these were considered to be good and very good in 82% and 80% of cases, respectively, regardless of age, prior nutritional status, and amount administered. Among the organoleptic characteristics of the formulas, the most influential factors were taste (82%), texture (77%), smell (74%), and the convenience of packaging and presentation (73%), with a positive influence on family satisfaction in 85.5% of those surveyed, with no correlation to age or nutritional status, but with a correlation to the amount of supplement received (less than 80% of TCI). Similar results were found with the use of HIF and HOFF, and a greater importance of taste and texture in the degree of satisfaction in the case of OPF (Figure 1).

When assessing the degree of adherence based on the amount of formula or supplement prescribed, this was higher than 75% intake of the amount indicated in 61% of patients, between 25% and 75% of the amount indicated in 26%, between 26% and 50% in 8%, less than 25% adherence to the amount prescribed in 5%, and data were similar in terms of the different types of ONS used (Table 5).

**Figure 2:**
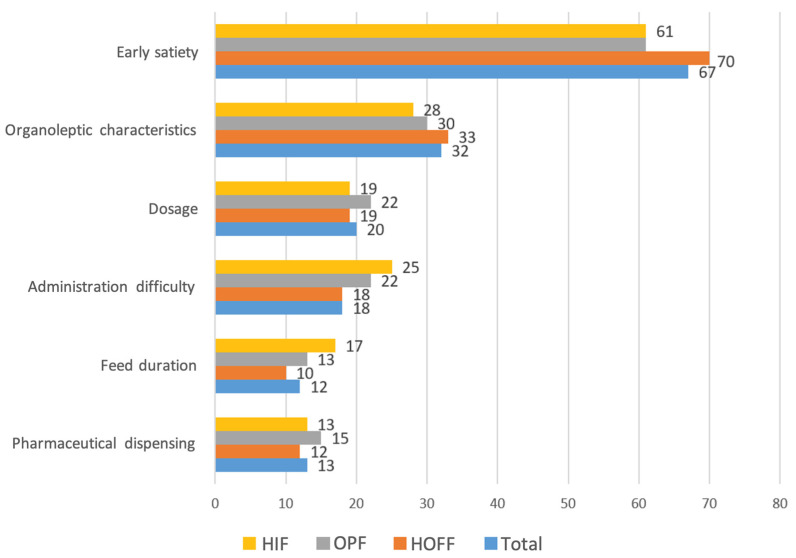
Factors associated with enteral nutrition non-adherence (percentage according to type of formula).

The smell of the formula was the organoleptic feature that most impacted non-adherence (55%), followed by taste and texture, and this was similar across all formulas used. The most influential factors for non-adherence were early satiety after administration (66.7%), organoleptic characteristics (32%), prescribed dose (20%), difficulties in authorising financing by medical inspectors of our health system in 18.4%, and duration of intake and dispensing problems in 13% of respondents (Figure 2).

**Figure 3:**
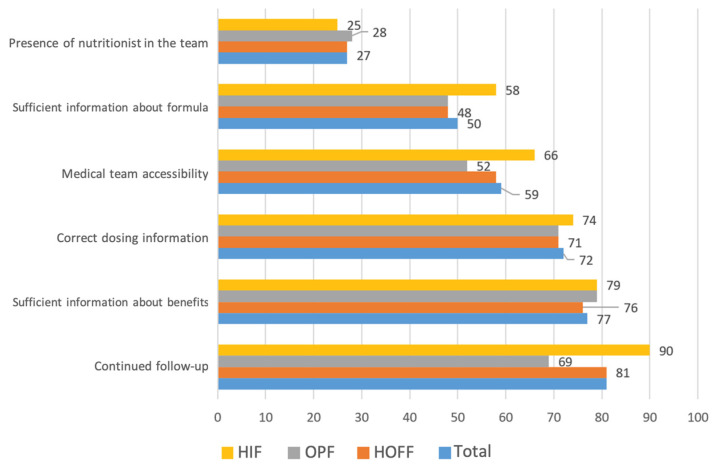
Factors associated with adequate enteral nutrition adherence (percentage according to type of formula).

The medical care-related factors that most impacted adequate adherence were sufficient information about the benefits of the supplements and form of use, at 77% and 72%, respectively, together with continued medical follow-up (81%) (Figure 3).

Chocolate (44%), followed by vanilla (37%), was the most widely accepted supplement flavour of all the formulations evaluated.

Of the benefits assessed, 83.5% of patients showed significant improvement in oral tolerance with the use of supplements, and improvement in different gastrointestinal symptoms such as diarrhoea, nausea, vomiting and abdominal pain (between 69% and 72% of patients, respectively).

With regard to the doctors surveyed, over 97% would prescribe the same indicated supplement or nutrition again, regardless of its type and formulation.

## 4. Discussion

There are currently few studies in the paediatric population on the perception of patient tolerance and acceptability that take into account the organoleptic properties of oral supplements [7]. The first similar study published in adult patients [8] found that much of the adherence to the administered treatment was due to the taste and smell of the formulas prescribed, which were therefore considered essential to ensure the efficacy of the treatment prescribed [8]. On this basis, and considering that these factors would be even more important in the paediatric population, given their particular characteristics, a second study that focused on this population was developed. PerceptiONS Jr is a study describing the perception of prescribing doctors, transmitted by the families of the children, with experience in the management of malnourished children regarding the nutritional solutions prescribed.

Disease-related malnutrition, as in the case of our patients, is a complex process in which nutritional status plays a key role in patient outcome [9] and significantly impacts patient morbidity and mortality, while also entailing a high cost of healthcare resources and expenses. Nutritional support is also associated with improved survival and decreased hospital admissions [10].

In view of the above, prescribing nutritional supplements in these children should occur on an individualised basis, taking into account both the underlying disease and the individual factors of each child and their environment, including their preferences. This makes the type of formula chosen especially important in terms of its composition and organoleptic properties, which, as we have discussed, are important factors that determine treatment adherence and, therefore, its short- and long-term efficacy.

Considering the importance of the above, we designed the PerceptiONS Jr study to determine patient degree of satisfaction and adherence as perceived by doctors, with information received from families. Among the prescribing doctors, the most common subspecialty was paediatric gastroenterology and nutrition in 87.7%, with this group of paediatricians primarily responsible for this task. For all of the included patients, both basic anthropometric and nutritional status assessments were performed using the Waterlow index, which allowed us to study different aspects, such as the amount of supplement administered, its characteristics, and the degree of adherence to the prescription according to nutritional status.

Of all the patients collected, patients under 6 months of age comprised the group with the lowest participation (20%), which should be taken into account when assessing the organoleptic characteristics of the formulations. As regards nutritional status in our series, most of our patients had mild acute malnutrition, which suggests supplementation was prescribed early on and is essential to the course of the disease [11].

The primary objective of the study was to evaluate tolerance to oral enteral formulas. In our case, 84% of patients used this route for supplementation, with a small percentage of cases in which the nutritional protocol was carried out through devices (NG tube, gastrostomy). These children were also significantly younger (<6 months). This age group also received the highest amount of formula with respect to their total calorie value during the day (47% received 100%). This may be because oral supplements at this age are more similar to their usual diet in the form of breast milk or formula. These data indicate the difficulty of assessing the organoleptic characteristics of the indicated formulas, though the degree of satisfaction with their use and adherence can be assessed in this sample from our series.

Multiple benefits perceived by the families were also observed in terms of improvement in mood and gastrointestinal symptoms, as reported in the PerceptiONS study in adults [8], and in vitality and perceived quality of life. This has already been postulated in several studies conducted in children for whom improved nutritional status resulted in improved quality of life for paediatric cancer patients [12,13]. Both the perceived improvement in overall condition of the child and their quality of life and achievement of the nutritional goal were significantly correlated to a greater amount of formula administered (*p* < 0.05). This shows that greater benefits were obtained with greater formula intake, and that therefore the patients’ nutritional improvement originated from its use.

With regard to the recorded benefits of nutritional treatment in the patients, 83.5% of the patients showed a significant improvement in the intake of foods other than the nutritional supplements and also in different gastrointestinal symptoms such as diarrhoea, nausea, vomiting and abdominal pain, with similar results in adult patients [8]. This datum suggests that, despite the widespread belief that supplement use decreases appetite and may ultimately hinder the administration and consumption of food by the oral route, improving nutritional status via these supplements nevertheless improves the possibility of increasing food intake by the same route.

In most of our patients, acceptance of the prescribed formulas was very good, with organoleptic characteristics being one of the most important conditioning factors. Among these factors, taste and texture were the most significant, with similar results found in the study conducted in adults [8]. In terms of flavour, chocolate was the best rated. Since this aspect has the greatest impact on acceptance and adherence in these patients, several studies have been conducted describing modulation of these children in terms of taste perception, which can be influenced by genetic and phenotypic factors, taste memory, and the patient’s clinical condition, all of which modify taste and, therefore, acceptance and adherence to the treatment prescribed [14]. No differences were found in the degree of satisfaction according to the formula prescribed, with high results for both polymeric formulas with fibre and peptide formulas. This provides important data as these formulas are typically more poorly accepted because of their taste, even as their use is necessary in patients with compromised gastrointestinal function, as they improve absorption, nutrient utilization, and gastrointestinal tolerance [15].

Another important factor assessed was the degree of adherence and compliance with the prescribed treatment, which was over 75%. This is in line with data published in 46 studies included in a systematic review conducted by Hubbard GP et al. [16], which assessed adherence in both inpatients and outpatients, reaching 74% and with various diseases. Studies conducted in non-hospitalised patients have found poorer treatment adherence (50%) due to the reduced monitoring of these patients as compared with studies conducted in a hospital setting [17]. They therefore suggest that optimised patient monitoring is essential to detect early problems that cause non-adherence and to resolve them early on.

As for the factors that most impacted adherence, organoleptic properties were the most significant compared with the number of doses prescribed. In the literature, high caloric density of formulas is correlated to better adherence, with this relationship being negative for prescribed treatment duration, though this has not been confirmed in subsequent studies [16].

In clinical practice, it should be taken into account that some factors related to medical care may condition adequate adherence to nutritional supplements. In our study, the most determining factors were adequate information about the benefits of supplements, how they were used, and continued medical monitoring. Therefore, to achieve adequate adherence, we must take care not only of aspects related to individual patient characteristics, but also aspects related to different aspects of our care, such as the time dedicated to explaining the indications and the different types of formulations, as well as ensuring adequate follow-up. In this regard, the addition of skilled nursing staff and experienced nutritionists could be helpful to reinforce and ensure said nutritional adherence.

On the other hand, among the organoleptic characteristics, the smell of the formula significantly impacted non-adherence (55%), followed by taste and texture in our series. Therefore, we must not only consider the taste of supplements, but also make an effort to research and improve the smell and texture of the different formulations offered in the industry and take them into account when prescribing them.

The factors associated with non-adherence were early satiety after consumption, the organoleptic characteristics, the prescribed dose, difficulties in authorising financing by inspection (this last one had not been described in previous studies), duration of intake and dispensing problems at the respondents’ pharmacies. As such, nutritional treatment compliance in our clinical practice is not only influenced by the characteristics of the formulations used, but also dosing-related aspects and administrative issues to be improved in many cases.

Given the above results, 97% of the doctors surveyed would prescribe the same nutritional therapy again, representing a positive perception on their part of the results obtained with prescription of enteral formulas. The same results were seen in the PerceptiONS study conducted in adult patients [8].

The PerceptiONS Jr study is the first paediatric study with a large sample size and is therefore representative of a clinical-reality-related nutritional supplement subscription and adherence. Our study assessed not only doctor’s perceptions about prescribing supplements to sick children, but also the degree of satisfaction among families and doctors, as well as factors that influenced adequate follow up of the indicated nutritional treatment. The results obtained show good satisfaction among the prescribing doctors in terms of patient tolerance and adherence to the formulas prescribed, independent of the formulas and of the previous nutritional status. The study describes patients of all ages and characteristics, which makes it extrapolatable to the Spanish paediatric population of children requiring nutritional support in an outpatient setting.

One of the limitations of the study is that the data are based on surveys with subjective opinions recorded by doctors and reported by families, which may not accurately reflect the patient experience. The study was conducted in Spanish public health hospitals, so extrapolation to other, less specialized, healthcare settings is difficult. On the other hand, no progress results have been collected via laboratory tests or successive nutritional assessments that objectively support some of the impressions collected by the respondents. Another limitation is that all of the doctors were invited to participate by Abbot, and that this might represent a limitation in the representativeness of the study. Even so, the high number of participants and the data referred to offer us a good opportunity to achieve the objectives established a priori. We therefore consider that further studies are needed to assess tolerance and factors influencing adherence to nutritional treatments in children, taking into account the impact that these factors may have on the course of the disease in these children.

## 5. Conclusions

In our study, the families surveyed perceived significant benefits from the prescribed formulas and supplements, with improvement in quality of life and the overall condition of the children, and adequate satisfaction, acceptability and adherence to treatment. The factors that most impacted treatment non-adherence were early satiety, organoleptic characteristics, particularly smell, and dosing amounts. The factors that positively impacted adherence were sufficient information about the characteristics of the formulations and their benefits, as well as continuity of care during follow up. Supplement administration significantly improves oral tolerance and different gastrointestinal symptoms.

In general, prescribing doctors would recommend the use of the indicated supplements again given the perceived benefits and nutritional and quality of life goals achieved.

Lastly, and considering the results of our study, we must continue to take into consideration, and research and improve, the organoleptic characteristics of nutritional formulas, as well as aspects related to their indication, individualized dosage, and clinical care, in order to ensure better nutritional adherence, and to ensure the achievement of nutritional goals and improvement in the clinical status of children.

## Figures and Tables

**Table 1:** Demographic and clinical characteristics of the surveyed professionals.

**Characteristics (*N* = 183)**	
**Age** **(median ± SD)**	39.6 ± 8.6 years
**Gender**	74% women/26% men
**Years of professional experience** **(median ± SD)**	10.6 ± 7.8 years
**Professional experience (%)**	
Paediatric gastroenterology/nutrition	88%
Paediatric neurology	1%
Paediatric oncology	4%
Other	7%

**Table 2:** Demographic characteristics of included patients.

	Total (N = 815)	HOFF (n1 = 525) (65%)	OPF (n2 = 127) (15%)	HIF (n3 = 163) (20%)
**Gender (% male)**	55%	56%	56%	51%
**Weight (kg)** **(median ± SD)**	17.6 ± 11.9 (2.3–74.8)	20.8 ± 11.61 (5.26–74.5)	19 ± 12 (17–58)	6.31 ± 2 (2.3–14.5)
**Height (cm)** **(median ± SD)**	105 ± 32.5 (46–179)	116 ± 28 (61–175)	108 ± 30 (103–179)	66 ± 10 (46.5–101.5)
**Waterlow index for weight** **(median ± SD)**	85.1 ± 12%	85 ± 13%	86.5 ±13.5%	85 ± 10%
**Acute malnutrition (% patients)**				
Mild	40%	41%	32%	40%
Moderate	25%	20%	21%	26%
Severe	7.4%	8%	7%	6%
**Waterlow index for height** **(median ± SD)**	93.6 ± 6.4%	93.53 ± 6.4%	94 ± 6.5%	94 ± 6%
**Chronic malnutrition (% patients)**				
Mild	35%	34%	33%	40%
Moderate	17%	17%	15%	20%
Severe	7%	8%	9%	4%

**Table 3:** Distribution by age of formula type, amount administered and administration route.

	Total (N = 815)	<6 Months (n1 = 62) (8%)	7–12 Months (n2 = 73) (9%)	>1–3 Years (n3 = 196) (24%)	>3–6 Years (n4 = 152) (19%)	>6–10 Years (n5 = 141) (17%)	>10 Years (n6 = 191) (24%)	Significance Level (Fisher’s Test)
**Distribution (%)**		7.6%	9%	24%	18.6%	17.4%	24.4%	
**Type of formula** HOFF OPF HIF								
	64%	-	8.2%	60%	79.6%	83.7%	84.8%	NS
	16%	-	6.8%	21%	19%	16.3%	15.2%	NS
	20%	100%	85%	19%	14%	-	-	0.001
**Quantity of formula relative to total calorie intake (%)**								
<30%	37.5%	9.7%	24.7%	37.7%	42%	43.5%	37.5%	NS
30–80%	48%	30.7%	52.5%	49.4%	48.6%	49.7%	48.2%	NS
80–99%	7%	12.6%	17.8%	7.6%	6%	2.6%	6.7%	NS
>100%	7.5%	47%	5.5%	5%	3.4%	4.2%	7.4%	0.001
**Route of administration**								
Oral	84%	74%	88%	83%	85%	84%	87%	NS
NG tube	5%	11%	1%	6%	5%	6%	4%	0.04
Gastrostomy/jejunostomy	6%	2%	4%	5%	8%	6%	5%	NS
Oral/NG tube	4%	13%	6%	3%	1%	4%	3%	NS
Oral/gastrostomy	1%	-	1%	3%	1%	-	1%	NS

**Table 4:** Percentage of good and very good satisfaction with the benefits observed by caregivers using formula.

	Total (N = 815)	HOFF (n1 = 525)	OPF (n2 = 127)	HIF (n3 = 163)
**Improvement in overall condition**	82%	79%	83%	88%
**Improvement in mood**	69%	66%	67%	86%
**Improvement in vitality/energy**	76%	74%	72%	83%
**Nutritional goal achieved**	80% *	77% **	79.5%	88%
**Improvement in patient quality of life**	79.5% *	77% **	79% #	88%
**Improvement in symptoms for which formula was prescribed**	77%	75% **	74% #	86%
**Interferes with daily intake of other foods**	26%	24%	22%	29%

Kruskal–Wallis test: * age under 6 months (*p* = 0.03 and *p* = 0.02); ** higher percentage of total calorie intake (TCI) (*p* = 0.014, *p* = 0.016 and *p* = 0.028); # older patient age (*p* = 0.017 and *p* = 0.015).

**Table 5:** Degree of treatment adherence and demographic and nutritional factors.

Degree of Adherence (% Total Volume/Daily)	<25%	25–50%	51–75%	>75%	Significance Level (Fisher’s Test)
Patients	38 (5%)	66 (8%)	215 (26%)	496 (61%)	-
Age					0.11
0–6 months (62)	2 (4%)	4 (6%)	13(21%)	43(69%)	
7–12 months (73)	4 (6%)	9 (12%)	13 (18%)	47 (64%)	
13–36 months (196)	10 (5%)	20 (10%)	51 (26%)	115 (59%)	
>3–6 years (152)	4 (3%)	14 (9%)	37 (24%)	97 (64%)	
>6–10 years (141)	10 (7%)	4 (3%)	36 (26%)	91 (64%)	
>10 years (191)	8 (4%)	15 (8%)	65 (34%)	103 (54%)	
Acute malnutrition					0.2
Mild (326)	20 (6%)	30 (9%)	73 (22%)	203 (62%)	
Moderate (205)	10 (5%)	17 (8%)	55 (27%)	123 (60%)	
Severe (60)	2 (3%)	2 (3%)	22 (37%)	34 (57%)	
Chronic malnutrition					0.6
Mild (285)	13 (5%)	26(9%)	69 (24%)	177 (62%)	
Moderate (139)	6 (4%)	8 (6%)	38 (27%)	87(63%)	
Severe (57)	2 (3.5%)	2 (3.5%)	21 (37%)	32(56%)	
Type of formula					0.22
HOFF (525)	30 (4%)	39 (7.5%)	151 (29%)	305 (58%)	
OPF (127)	3 (3%)	11 (9%)	31 (24%)	82 (65%)	
HIF (163)	5 (3%)	16 (10%)	33 (20%)	109 (67%)	

## Data Availability

The original contributions presented in the study are included in the article, further inquiries can be directed to the corresponding author.

## Abbreviations

HIF: hyper-caloric infant formula (Similar High Energy), HOFF: hyper-caloric oral formula with fibre (Pediasure Plus Fibre), ONS: oral nutritional supplement, OPF: oral peptide formula (Pediasure Peptide), TCI: total calorie intake (%).

## Appendix A. List of PerceptiONS Jr Group Members (in Alphabetical Order)

Algar Serrano M (H. Universitari Doctor Josep Trueta. Girona), Altali Alhames K (H. Universitario Móstoles. Madrid), Alvarado Cárcamo BA (H. Sant Joan de Deu. Barcelona), Arcos Machancoses JV (H. Clínico Universitario Valencia. Valencian Community), Balboa Vega MJ (Complejo H. Virgen Macarena. Seville. Andalusia), Balmaseda Serrano E (Complejo H. Universitario de Albacete: Castilla-La Mancha), Barahona Rondón L (H. Universitario Infanta Leonor. Madrid), Barrio Torres J (H. Universitario Fuenlabrada. Madrid), Barros García P (H. Universitario Clínico San Cecilio. Granada. Andalusia), Benavente García JJ (University Hospital Complex of Cartagena. Murcia), Benítez Moscoso G (H. General Juan Ramon Jiménez. Huelva. Andalusia), Bergua Martínez A (Complejo H. Universitario La Paz. Madrid), Blanco Rodríguez M (H. Universitario Fundación Jiménez Diaz. Madrid), Cabello Ruiz V (Complejo H. Vall D’ Hebrón. Barcelona. Catalonia), Canals Baeza A (H. Universitario Sant Joan D’Alacant. Alicante. Valencian Community), Cañedo Villarroya E (H. Universitario Niño Jesús. Madrid), Carreira Sande N (Complejo H. Universitario Santiago. Santiago de Compostela. Galicia), Cerchi Fernández AC (H. Clínico Universitario Virgen de La Arrixaca. Murcia), Correcher Medina P (H. La Fe Complex. Valencia. Valencian Community), Cossio Lanzari E (H. Clínico Universitario Virgen de La Arrixaca. Murcia), Costantini Valletta F (H. Universitario Móstoles. Madrid), Cuadrado Martín S (H. General Universitario Nuestra Señora del Prado. Talavera de la Reina. Castilla- La Mancha), Cuevas Moreno A (Complejo H. Vall D’ Hebrón. Barcelona. Catalonia), de la Mano Hernández A (H. Universitario Niño Jesús. Madrid), de los Santos Mercedes M (H. de Sant Joan Despi Moises Broggi. Barcelona. Catalonia), Díaz Molina G (H. de Manises. Valencia. Valencian Community), Diez Vela E (H. Universitario Príncipe de Asturias. Alcalá de Henares. Madrid), Domenech Marce E (H. Sant Joan de Deu. Barcelona. Catalonia), Donado Palencia P (H. General Universitario de Ciudad Real. Castilla-La Mancha), Donat Aliaga E (Complejo. H. La Fe. Valencia. Valencian Community), Egea Castillo N (H. de Sant Joan Despi Moises Broggi. Barcelona. Catalonia), Escartín Madurga L (H. Clínico Universitario Lozano Blesa de Zaragoza: Aragón), Fernández Antuña L (Fundación H. Sant Pau i Santa Tecla. Tarragona. Catalonia), Fernández Ventureira V (H. Universitario Joan XXIII. Tarragona. Catalonia), Ferrando Mora M (H. Universitario Sant Joan D’Alacant. Alicante. Valencian Community), Ferrer González P (Complejo H. la Fe. Valencia. Valencian Community), Flores Pérez P (H. Universitario De La Princesa. Madrid), Gallardo Padilla M (H. Universitario Infanta Elena. Valdemoro. Madrid), García Arena L (H. Parc Sanitari Sant Joan de Deu. Barcelona. Catalonia), García Aldana D (Complejo H. Reina Sofía. Córdoba. Andalucía), García Arenas D (H. Parc Sanitari Sant Joan de Deu. Barcelona. Catalonia), García Barba S (H. Universitario Fuenlabrada. Madrid), García Díaz A (H. Universitario Puerta de Hierro Majadahonda. Madrid), García Hidalgo L (Complejo H. Regional Universitario Malaga. Andalusia), García Jiménez A (Complejo H. Universitario La Paz. Madrid), García MugaI (Complejo H. San Pedro La Rioja), García Peris M (Complejo H. la Fe. Valencia. Comunidad Valenciana), García Volpe C (H. De Sant Joan Despi Moises Broggi. Barcelona. Catalonia), García-Ribera Ruiz S (H. del Mar. Barcelona. Catalonia), Gascón Galindo C (H. Universitario Ramon y Cajal. Madrid), Germán Diaz M (Complejo H. Universitario 12 de Octubre. Madrid), Gilbert Pérez JJ (Complejo H. Reina Sofia. Córdoba. Andalusia), González Vives L (H. Universitario Infanta Leonor. Madrid), González de Caldas Marchal R (Complejo H. Reina Sofia. Córdoba. Andalucía), González Espín AI (Complejo H. Jaén. Andalusia), Guevara Caviedes LN (Consorcio Sanitari De Terrassa H. de Terrassa. Barcelona. Catalonia), Guillen Rey N (H. Universitari Sant Joan de Reus. Catalonia), Gutiérrez Sánchez A (H. Parc Sanitari Sant Joan de Deu. Barcelona. Catalonia), Gutiérrez Vilar M (Complejo H. Nuestra Señora Candelaria-Ofra. Santa Cruz de Tenerife. Canary Islands), Ibarra Rubio M (H. Universitari Mutua Terrassa. Barcelona. Catalonia), Izquierdo Martin A (Complejo H. Universitario Badajoz. Extremadura), Jiménez Muñoz M (Complejo H. Regional Universitario Málaga. Andalusia), Ribelles AJ (Complejo H. La Fe. Valencia. Valencian Community), Largo Blanco EM (Consorcio H. General Universitario Valencia. Valencian Community), Llerena Santa Cruz E (H. Universitario Joan XXIII. Tarragona. Catalonia), Llopis Lera M (H. De Sant Joan Despi Moises Broggi. Barcelona. Catalonia), López Casado MA (H. General Universitario Virgen de las Nieves. Granada. Andalusia), López Liñán MJ (Consorcio anitari De Terrassa H. de Terrassa. Barcelona. Catalonia), López Sánchez MA (H. Universitario de Poniente. Almería. Andalusia), López Sánchez B (Complejo H. Toledo. Castilla-La Mancha), López-Seoane Puente FJ (H. General Universitario San Carlos. Madrid), Lorite Cuenca R (Complejo H. Vall D’ Hebron. Barcelona. Catalonia), Luque Pérez S (H. Punta de Europa. Cádiz. Andalusia), Madrid Rodríguez A (Complejo H. Regional Universitario Malaga. Andalusia), Manzano Infante MJ (Complejo H. Nuestra Señora de Valme. Seville. Andalusia), Marcilla Vázquez C (Complejo H. Universitario Albacete. Castilla-La Mancha), Marco Hernández AM (H. Universitario Doctor Peset. Valencia. Valencian Community), Martín Rivada A (Complejo Hosp. Canary Islands University. Santa Cruz de Tenerife. Canary Islands), Martín Masot R (Complejo H. Regional Universitario Málaga. Andalusia), Martínez Chicano D (H. de Sant Joan Despi Moises Broggi. Barcelona. Catalonia), Martínez Pastor N (H. General Universitari D’Alacant. Alicante. Valencian Community), Martínez Pérez J (H. Universitario Niño Jesús. Madrid), Martínez Villar M (Hosp. Universitario Puerta de Hierro Majadahonda. Madrid), Martinón Torres N (Complejo H. Universitario Santiago. Santiago de Compostela. Galicia), Medina González M (H. General Universitario Virgen de las Nieves. Granada. Andalucía), Mesonero Cavia S (Consorcio Sanitari De Terrassa H. de Terrassa. Barcelona. Catalonia), Millán Jiménez A (Complejo H. Nuestra Señora de Valme. Seville. Andalusia), Miralles Llumà L (H. Parc Sanitari Sant Joan de Deu. Barcelona. Catalonia), Montero Valladares C (Complejo H. Virgen Del Rocío. Seville. Andalusia), Montes Arjona AM (H. Universitario del Henares. Madrid), Moráis López A (Complejo H. La Paz. Madrid), Muñoz Martínez P (H. Rafael Méndez de Lorca. Murcia), Murcia Clemente L (H. Universitario de Vinalopo. Alicante. Valencian Community), Navarro Martínez M (H. Universitario Torreveja. Alicante. Valencian Community), Nova Lozano C (H. Clínico Universitario Valencia. Valencian Community), Olivas Mazón R (H. Clínico Universitario Valencia. Valencian Community), Ortega Magán E (H. General Universitario Gregorio Marañón. Madrid), Oya Magadán I (H. de Sant Joan Despi Moises Broggi. Barcelona. Catalonia), Palomino Pérez LM (H. Universitario Niño Jesús. Madrid), Peláez Pleguezuelos I H. General Universitario Virgen de las Nieves. Granada. Andalusia), Pena Lamelas FJ (H. de la Vega Lorenzo Guirao. Cieza. Murcia), Peñafiel Freire DM (Complejo H. Navarra. Navarre), Pérez Aragón C (H. Universitario Puerta del Mar. Cádiz. Andalusia), Pérez Requena N (H. Parc Sanitari Sant Joan de Deu. Barcelona. Catalonia), Pérez Vera M (H. de Mérida. Extremadura), Pizarro Ruiz M (Complejo H. Cartagena. Murcia), Plácido Paias R (H. de Mérida. Extremadura), Quiroga De Castro A (H. Universitario Puerta del Mar. Cádiz. Andalusia), Raya Muñoz J (Complejo H. Vall D’ Hebron. Barcelona. Catalonia), Redecillas Ferreiro S (Complejo H. Vall D’ Hebrón. Barcelona. Catalonia), Resa Serrano E (H. General la Mancha Centro. Castilla- La Mancha), Rivero de la Rosa MC (Complejo H. Virgen Macarena. Seville. Andalusia), Rodríguez López S (H. de Jerez. Cádiz. Andalusia), Rodríguez Martínez A (Complejo H. Virgen del Rocío. Seville. Andalusia), Rodríguez Sánchez A (Complejo H. Cartagena. Murcia), Rodríguez Martín LE (Complejo H. Nuestra Señora de Valme. Seville. Andalusia), Rodríguez Salas M (Complejo H. Reina Sofía. Córdoba. Andalusia), Rojas de González N (H. Comarcal La Línea de la Concepción. Cádiz. Andalusia), Romero García C (H. Infanta Elena. Madrid), Romero Pérez S (H. General Básico Minas De Riotinto. Huelva. Andalusia), Rosell Camps A (H. Universitario Son Espases. Mallorca. Balearic Islands), Ruiz Tudela L (H. Rafael Méndez de Lorca. Murcia), Salcedo Mora X (H. Recoletas Campo Grande. Valladolid. Castilla León), Salgado Valencia S (H. Universitario rio Hortega. Valladolid. Castilla León), Salvador Pinto T (H. Marina Baixa. Alicante. Valencian Community), San Miguel López L (H. Universitario Príncipe de Asturias. Alcalá de Henares. Madrid), Sánchez López MA (H. General Universitario Gregorio Marañón. Madrid), Sánchez Pérez M (H. Universitario Costa del Sol. Málaga. Andalusia), Sánchez Rodríguez I (H. Universitario Móstoles. Madrid), Sarto Guerri B (Complejo H. Vall D’ Hebron. Barcelona. Catalonia), Serra Font S (H. de la Santa Creu i Sant Pau. Barcelona. Catalonia), Simó Jordá R (H. Universitario Doctor Peset. Valencia. Valencian Community), Suarez Fuentes T (Fundación H. Sant Pau i Santa Tecla. Tarragona. Catalonia), Suriñach Ayats B (H. de la Santa Creu i Sant Pau. Barcelona. Catalonia), Valero Pérez G (H. de Torrejón. Madrid), Valverde Fernández J (Complejo H. Virgen del Rocío. Seville. Andalusia), Vázquez Gómez JA (Complejo H. San Pedro. La Rioja), Vecino López V (H. Universitario San Carlos. Madrid), Velasco Rodríguez-Belvís M (H. Universitario Niño Jesús. Madrid), Vicente Quesada A (H. del Mar. Barcelona. Catalonia), Violadé Guerrero F (Complejo H. Virgen del Rocío. Seville. Andalusia).
